# Proton-Coupled Electron Transfer at the Surface of Polyoxovanadate-Alkoxide Clusters

**DOI:** 10.1021/acs.accounts.3c00166

**Published:** 2023-06-06

**Authors:** Kathryn R. Proe, Eric Schreiber, Ellen M. Matson

**Affiliations:** Department of Chemistry, University of Rochester, Rochester, New York 14627, United States

## Abstract

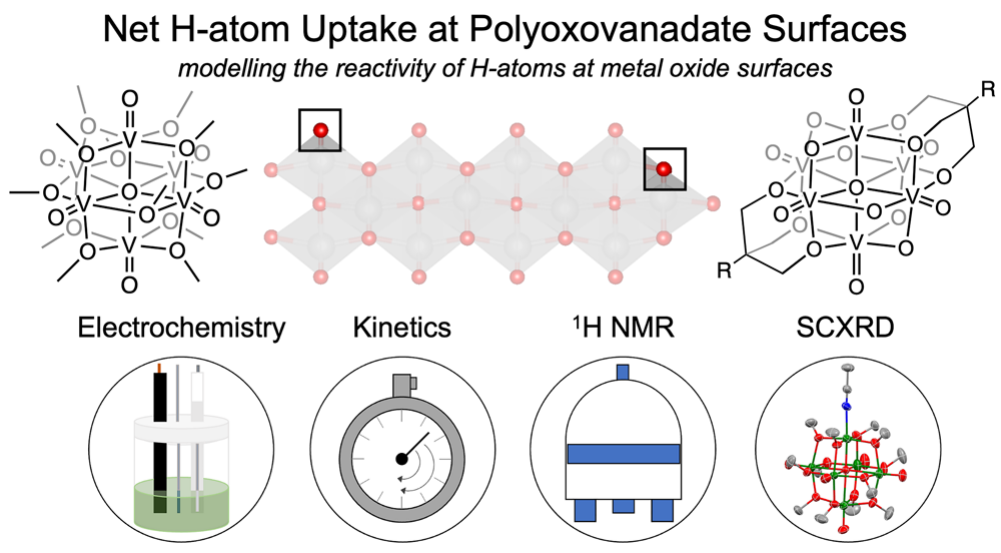

Proton-coupled electron transfer (PCET) is a fundamental process involved in all areas of chemistry, with relevance to biological transformations, catalysis, and emergent energy storage and conversion technologies. Early observations of PCET were reported by Meyer and co-workers in 1981 while investigating the proton dependence of reduction of a molecular ruthenium oxo complex. Since that time, this conceptual framework has grown to encompass an enormous scope of charge transfer and compensation reactions. In this Account, we will discuss ongoing efforts in the Matson Laboratory to understand the fundamental thermodynamics and kinetics of PCET processes at the surface of a series of Lindqvist-type polyoxovanadate clusters. This project aims to provide atomistic resolution of net H atom uptake and transfer at the surfaces of transition-metal oxide materials.

First, we discuss our efforts aimed at understanding PCET at metal oxide surfaces using the Lindqvist-type polyoxovanadate-alkoxide (POV-alkoxide) cluster [^*n*^Bu_4_N]_2_[V_6_O_13_(TRIOL^NO_2_^)_2_]. These clusters reversibly bind H atom equivalents at bridging oxide sites, mirroring the proposed uptake and release of e^–^/H^+^ pairs at transition-metal oxide surfaces. Summarized results include the measurement of bond dissociation free energies of surface hydroxide moieties (BDFE(O–H)) as well as mechanistic analyses that verify concerted proton electron transfer as the operative pathway for PCET at the surface of POV-alkoxide clusters.

Next, we discuss net proton and H atom uptake at the surface of reduced variants of the Lindqvist-type POV-alkoxide cluster, [V_6_O_7_(OR)_12_]^*n*^ (R = Me, Et; *n* = −2, −1, 0, + 1). In the case of these low-valent POV-alkoxide clusters, nucleophilic bridging sites are kinetically inhibited by functionalization of the cluster surface with organic ligands. This molecular modification enables site-selectivity in proton and H atom uptake to terminal oxide sites. The impact of reaction site and cluster electronics on reaction driving force of PCET is explored, with core electron density playing a critical role in dictating thermodynamics of H atom uptake and transfer. Additional work described here contrasts the kinetics of PCET at terminal oxide sites to the reactivity observed at bridging oxides in POV-alkoxide clusters.

Overall, this Account summarizes our foundational knowledge regarding the assessment of PCET reactivity at the surfaces of molecular metal oxides. Drawing analogies between POV-alkoxide clusters and nanoscopic metal oxide materials provide design principles for the advancement of materials applications with atomic precision. These complexes are additionally highlighted as tunable redox mediators in their own right; our studies demonstrate how cluster surface reactivities can be optimized by modifying electronic structure and surface functionalities.

## Key References

- FertigA. A.; MatsonE. M.Connecting Thermodynamics and Kinetics of Proton Coupled Electron Transfer at Polyoxovanadate Surfaces Using the Marcus Cross Relation. Inorg. Chem.2023, 62, 1958–196710.1021/acs.inorgchem.2c0254136049052PMC9906739.^1^ This study discusses reversible 6e^–^/6H^+^ uptake at bridging-oxide sites at the surface of a polyoxovanadate-alkoxide cluster. The BDFE(O–H)_avg_ is determined electrochemically, and the reactivity of the reduced species toward O_2_ reduction is explored.
- FertigA. A.; BrennesselW. W.; McKoneJ. R.; MatsonE. M.Concerted Multiproton-Multielectron Transfer for the Reduction of O_2_ to H_2_O with a Polyoxovanadate Cluster. J. Am. Chem. Soc.2021, 143 ( (38), ), 15756–1576810.1021/jacs.1c0707634528799.^2^ This publication evaluates different analytical approaches to measuring O–H bond free energies using a variety of electrochemical and chemical approaches. The driving force of this multielectron/multiproton PCET is related to the rate constant of H atom transfer through the Marcus cross relation.
- SchreiberE.; BrennesselW. W.; MatsonE. M.Charge-State Dependence of Proton Uptake in Polyoxovanadate-alkoxide Clusters. Inorg. Chem.2022, 61, 4789–480010.1021/acs.inorgchem.1c0293735293218PMC8965876.^3^ This study develops an understanding of acid-induced terminal defect formation by probing the influence of electronic structure of the surface basicity of organosaturated polyoxovanadate-alkoxide clusters.
- SchreiberE.; FertigA. A.; BrennesselW. W.; MatsonE. M.Oxygen-Atom Defect Formation in Polyoxovanadate Clusters via Proton-Coupled Electron Transfer. J. Am. Chem. Soc.2022, 144, 5029–504110.1021/jacs.1c1343235275632PMC8949770.^4^ This publication examines the thermochemistry and mechanism of the reduction of terminal oxido moieties of polyoxovanadate-alkoxides by proton-coupled electron transfer.

## Introduction

**Scheme 1:**
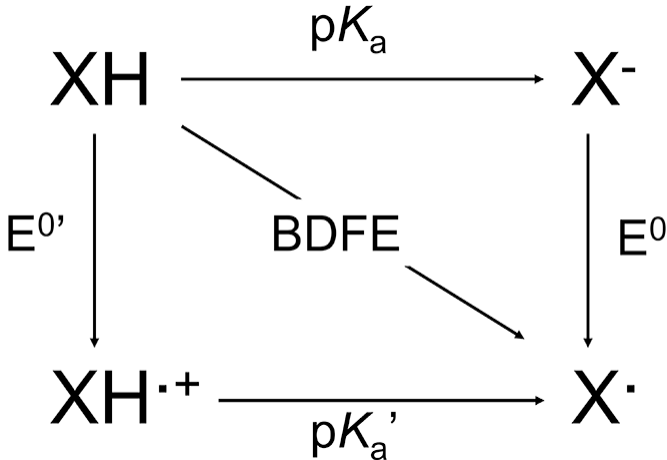
A Generalized Square Scheme Depicting Individual Electron and Proton Transfer Events That Make up a PCET reaction, with Their Respective Thermochemical Values (i.e. *E*^0^ and p*K*_a_) Concerted electron/proton transfer from XH is represented by the reaction on the diagonal. The thermochemistry of X–H bond scission is defined throughout our work by the BDFE of the X–H bond.

The rich electrochemical properties of transition-metal oxides have positioned these materials as valuable mediators in emergent energy-related technologies (e.g., fuel cells,^5^ optoelectronic devices,^6^ photo/electrochemical catalysts^7^). In all these applications, a defining feature of active materials is their ability to accept and transfer H atom equivalents (i.e., proton/electron pairs) via proton-coupled electron transfer (PCET). The mechanism(s) of these processes have been interrogated by both empirical and computational methods, providing evidence for both concerted proton-electron transfer (CPET) and electron transfer-proton transfer (ET-PT) pathways (Scheme 1). However, further progress in performance optimization via materials design requires an improved understanding of thermal and kinetic considerations that dictate the reactivity of H atom equivalents at the surface of transition-metal oxides.

PCET reactivity mediated by transition-metal oxides is governed by thermodynamics, where the difference in energies of bonds broken and formed influences reaction equilibria.^8−10^ Further progress in predicting the thermochemistry of O–H bond formation and cleavage at metal oxide surfaces requires improved atomistic insight into the nature of reactive sites, and the role local structures play in influencing bond dissociation free energy (BDFE(O−H)) values.^11^ To this end, valuable information has been gained from theoretical investigations. Several reports have outlined the optimized structures of H atoms bound to oxides, providing insight into preferred binding sites at various H atom doping levels.^12^ Direct experimental insight into the reactivity of H atom equivalents at metal oxide surfaces has been hampered by the complexity of bulk transition-metal oxides. Indeed, the extended structure of these materials results in competing H atom intercalation^13^ and the presence of defect sites that alter mechanisms of PCET postulated for the pristine assembly.^14^ To circumvent these challenges, the study of molecular models has become an attractive route to both understand reactivity and bonding as well as uncover design criteria for subsequent material development.^15,16^

Polyoxometalates (POMs) stand out as a particularly intriguing family of compounds for understanding PCET at metal oxide surfaces.^17^ Composed of multiple transition-metal centers linked together through bridging oxygen atoms, these molecular metal oxides exist on the continuum between small molecules and extended solids. Of relevance to PCET in metal oxide materials, POMs feature reduction potentials that are sensitive to acid concentration, indicating net H atom uptake under reducing conditions.^2,18,19^ Notably, the molecular structure of POMS limits all reactivity with protons to the surface of the complex, poising POMs to serve as efficient PCET mediators by eliminating kinetic barriers associated with lattice intercalation of H atoms.

**Figure 1:**
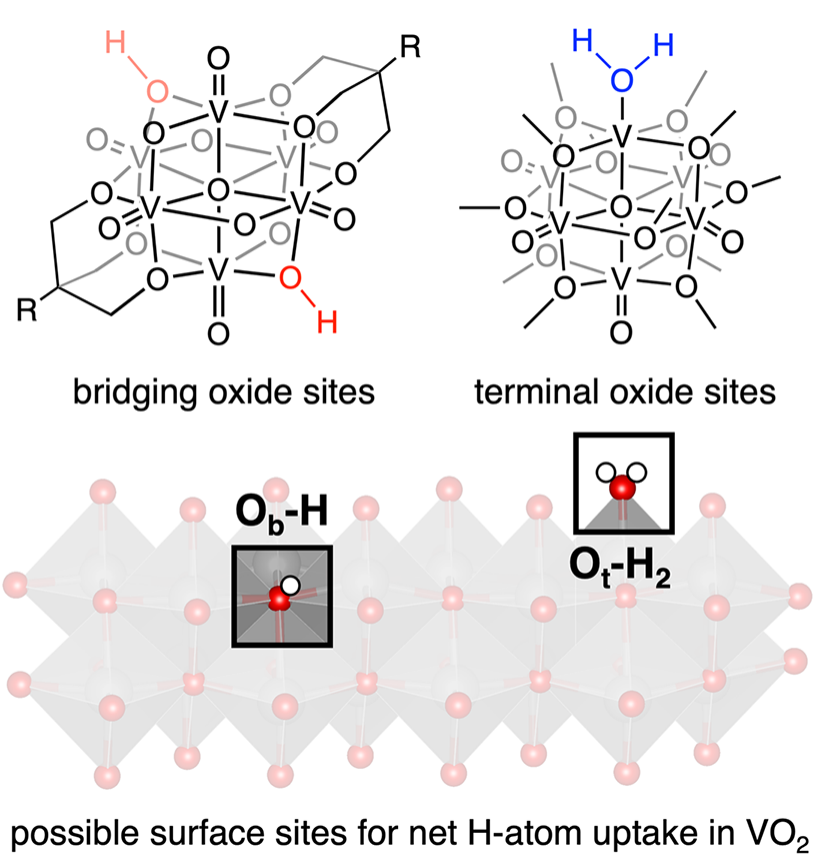
Net H atom uptake at polyoxovanadate-alkoxide surfaces described in this work (top), which provide thermodynamic and kinetic insights into similar reactivity at metal oxide surfaces (bottom). While a variety of other H atom uptake sites are possible in materials, we highlight the binding modes for which we have generated cluster analogues.

A distinct subclass of POMs is the Lindqvist-type polyoxovanadate-alkoxide (POV-alkoxide) cluster: [V_6_O_19–*x*_(OR)_*x*_]^−*n*^ (Figure 1). These hexavanadate assemblies differ from canonical POMs in that the high charge density of the small [V_6_O_19_]^−8^ core destabilizes this structure in the absence of bridging ligands.^20^ Decreasing the charge of oxide linkers by organofunctionalization stabilizes composite V centers in a variety of oxidation states (e.g., V^III^, V^IV^, V^V^), facilitating access to a range of core electronic structures.^21^ Additionally, the diverse range of ligands reported in POV-alkoxide structures provide opportunities to investigate the role surface ligands play in influencing the physicochemical properties of nanoscopic metal oxides.^21^

This Account is a summation of the work our group has performed over the past 4 years building an understanding of PCET at the surface of POV-alkoxide clusters. Our work with high- and low-valent derivatives of these hexavanadate assemblies has allowed our team to elucidate factors that influence net H atom uptake and transfer at bridging and terminal-oxide sites.^1−4,16,22,23^ Efforts described here outline various approaches for BDFE(O–H) determination in molecular cluster complexes.^1,2,4,23^ We also summarize a series of thermodynamic and kinetic investigations that reveal details about the mechanism of PCET at metal oxide surfaces, with particular attention to the role oxide position plays in dictating rate and activation parameters of net H atom uptake.^1,2,4,23^ Our group’s approach to probing and comparing the thermal and kinetic factors that dictate PCET at POV-alkoxides provides atomistic insights into similar reactivity in bulk and nanocrystalline material surfaces. More importantly, this work has laid a foundation for the development of a unique class of efficient catalysts for PCET-mediated small molecule transformations.

### PCET at Bridging Oxide Ligands in Polyoxovanadates

The interaction of reduced POMs with protons has been recognized for decades, noted most clearly in the substantial differences in electrochemical profiles of the metal oxide assemblies in neutral and acidic environments. In the absence of protons, most POMs exhibit 1e^–^ reduction events. Addition of acid results in anodic shifts of these electrochemical processes; in some cases, individual redox processes appear to merge into multielectron/multiproton transfer events. To date, the majority of work has focused on leveraging multielectron reactivity in POMs for applications in energy storage devices.^24^ The primary focus on performative metrics has resulted in comparatively less work emphasizing an understanding of PCET reactivity at the surface of POMs.

A 2015 study by Sami and co-workers was the first example of the explicit determination of a BDFE(O–H) for a reduced POM.^25^ The authors chose to investigate the divanadium-substituted polyoxotungsate cluster, [PV_2_W_10_O_40_]^−5^. The incorporation of vanadate ions into the tungsten assembly increases the basicity of the cluster surface, directing proton uptake to the bridging oxide moieties located between vanadium ions. Through the use of the Bordwell equation (eq 1), the BDFE(O–H) for 1e^–^/1H^+^ reduced assembly, [PV^V^V^IV^W_10_O_39_(OH)]^−5^, was determined to be 75.4 kcal mol^–1^.
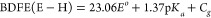
1

**Scheme 2:**
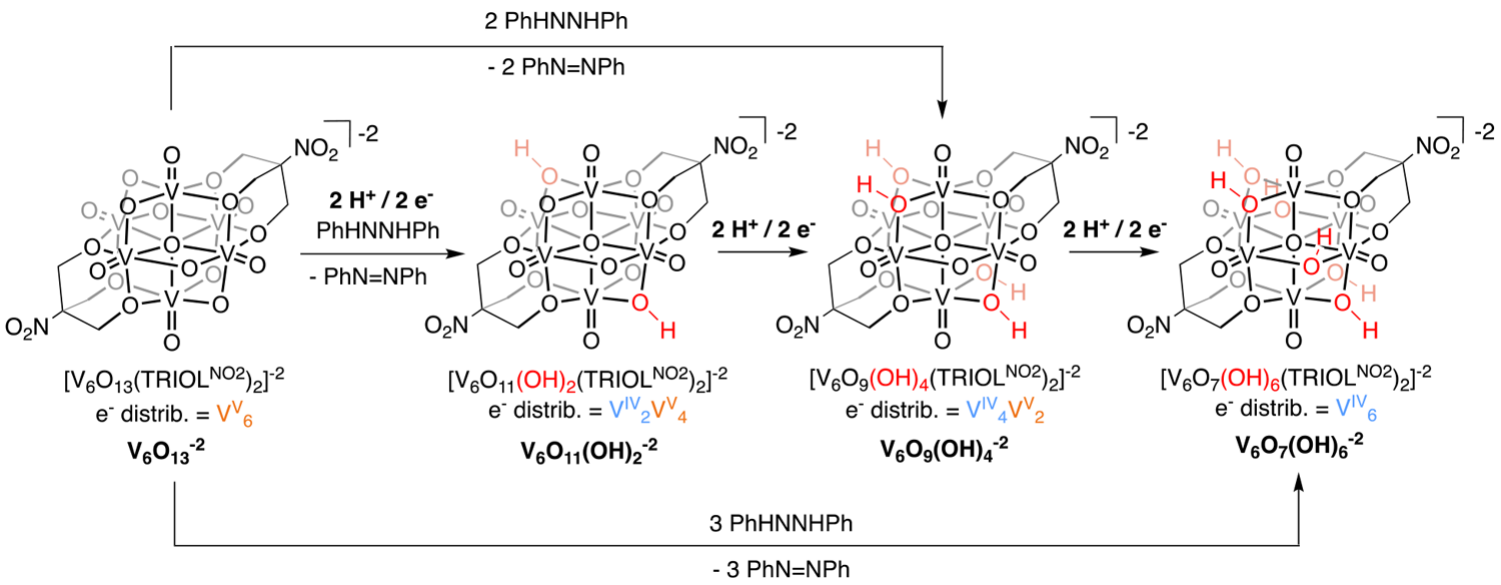
Reduction of **V**_**6**_**O**_**13**_^**–2**^ via PCET and Formation of [V^IV^_2_V^V^_4_O_11_(OH)_2_(TRIOL^NO_2_^)_2_]^−2^ (**V**_**6**_**O**_**11**_**(OH)**_**2**_^**–2**^), **V**_**6**_**O**_**9**_**(OH)**_**4**_^**–2**^ and **V**_**6**_**O**_**7**_**(OH)**_**6**_^**–2**^

Inspired by this study, our research team set out to establish a standard approach for determining BDFE(O–H) values of reduced POMs. Our initial investigation focused on H atom uptake in a series of high-valent POV-alkoxide clusters, [*^n^*Bu_4_N]_2_[V_6_O_13_(TRIOL^NO2^)_2_] (**V**_**6**_**O**_**13**_^**–2**^; TRIOL^NO_2_^ = (OCH_2_)_3_CNO_2_).^26^ Previous work by Zubieta and co-workers had demonstrated the ability of a similar cluster, [V_6_O_13_(TRIOL^Me^)_2_]^−2^ (TRIOL^Me^ = (OCH_2_)_3_CCH_3_), to access a variety of reduced states through net 2e^–^/2H^+^ addition reactions (e.g., [V^IV^_2_V^V^_4_O_11_(OH)_2_TRIOL^Me^)_2_]^−2^, [V^IV^_4_V^V^_2_O_9_(OH)_4_TRIOL^Me^)_2_]^−2^, and [V^IV^_6_O_7_(OH)_6_TRIOL^Me^)_2_]^−2^).^1,2^ In all examples, H atom uptake occurs at bridging oxido ligands. Our team opted to investigate the nitro-substituted variant of the POV-alkoxide cluster due to its modest reduction potential and improved crystallinity.^1^ Comparable reactivity of **V**_**6**_**O**_**13**_^**–2**^ with H atom equivalents was observed, as outlined in Scheme 2. Formation of the reduced complexes was confirmed by electronic absorption spectroscopy and electrospray ionization mass spectrometry (ESI-MS). Additionally, structural characterization of the 4e^–^/4H^+^ ([V_6_O_9_(OH)_4_(TRIOL^NO_2_^)_2_]^−2^; **V**_**6**_**O**_**9**_^**–2**^) and 6e^–^/6H^+^ ([V_6_O_7_(OH)_6_(TRIOL^NO_2_^)_2_]^−2^; **V**_**6**_**O**_**7**_^**–2**^) reduced complexes was possible via single crystal X-ray diffraction.

At the outset of our studies, we sought to determine thermochemistry of proton and electron transfer to the parent POV-alkoxide cluster, **V**_**6**_**O**_**13**_^**–2**^. Our original plan relied on our ability to measure individual electron transfer and proton transfer steps. While isolation of the reduced form of the POV-alkoxide cluster, [V_6_O_13_(TRIOL^NO_2_^)_2_]^−3^, was straightforward, experiments targeting protonation of the Lindqvist ion proved difficult. These experimental challenges translated to significant barriers associated with the determination of BDFEs of the reduced variants of the POV-alkoxide cluster.

Initial studies to elucidate BDFE(O–H)_avg_ of the bridging hydroxide moieties at the surface of **V**_**6**_**O**_**13**_^**–2**^ employed an elegant method reported by Dempsey and co-workers.^27^ This strategy invokes the construction of a “Pourbaix” diagram adapted for nonaqueous systems. Reduction potentials of redox active complexes are measured in the presence of a series of organic acids with variable p*K*_a_ values. Plotting the reduction potential vs p*K*_a_ of the added organic acid results in a diagram which allows thermochemical information describing proton and electron transfer to be obtained for unstable intermediates.

**Figure 2:**
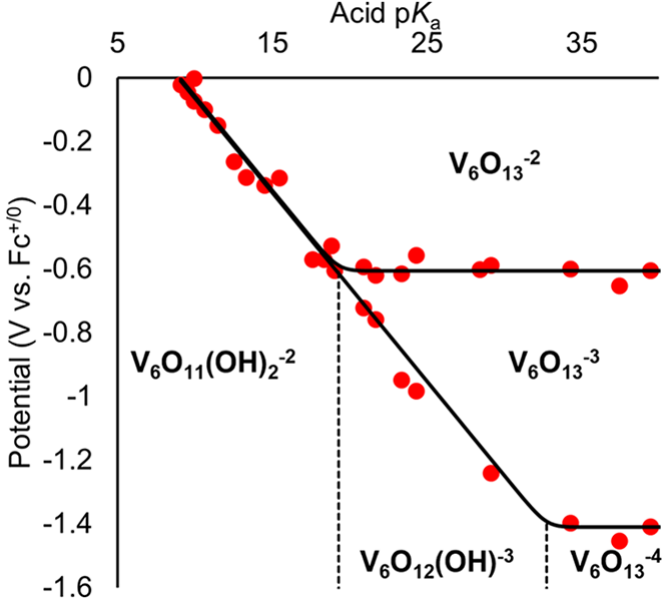
Potential p*K*_a_ diagram for **V**_**6**_**O**_**13**_^**–2**^ describing reduction potentials (red dots) in the presence of organic acids and 100 mM [^*n*^Bu_4_N][PF_6_]. Figure reproduced with permission from ref (1). Copyright 2023 American Chemical Society.

The cyclic voltammogram (CV) of **V**_**6**_**O**_**13**_^**–2**^ was analyzed in the presence of organic acids with p*K*_a_ values ranging from 9.1 to 39.5 (Figure 2). Upon addition of organic acids with p*K*_a_ values greater than 33, the electrochemical profile of **V**_**6**_**O**_**13**_^**–2**^ remained largely unchanged from that observed in the absence of protons, consisting of two one-electron reduction events.^2^ In the presence of organic acids with p*K*_a_ values ranging between 19 and 33, loss of reversibility and anodic shift of the second reduction event were observed. Under these experimental conditions, *E*_1/2_ values for irreversible redox events were determined through evaluation of the second derivative of the CV.^2^ In the case of CVs run of **V**_**6**_**O**_**13**_^**–2**^ in the presence of organic acids with p*K*_a_ values <19, the two distinct one-electron reduction events collapse into a single, two-electron redox process. The slope of ∼60 mV/p*K*_a_, calculated by linear regression for the portion of our potential-p*K*_a_ diagram from p*K*_a_ 9–19, indicates a 1:1 ratio of protons to electrons transferred which we can attribute the 2e^–^/2H^+^ transfer between **V**_**6**_**O**_**11**_**(OH)**_**2**_^**–2**^ and **V**_**6**_**O**_**13**_^**–2**^.

The construction of a potential-p*K*_a_ diagram allows for the approximation of the p*K*_a_ values of protonated and reduced forms of **V**_**6**_**O**_**13**_^**–2**^ (**V**_**6**_**O**_**11**_**(OH)**_**2**_^**–2**^ and [V_6_O_12_(OH)(TRIOL^NO_2_^)_2_]^−3^). In Figure 2, the points where acid-independent redox events (horizontal lines) intersect acid-dependent redox events (diagonal line) indicate the basicity of the cluster is sufficient to deprotonate the acid present in solution. This data shows that the reduced forms of **V**_**6**_**O**_**13**_^**–2**^ are quite basic, with p*K*_a_ values of 19.3 and 32.7 for **V**_**6**_**O**_**11**_**(OH)**_**2**_^**–2**^ and [V_6_O_12_(OH)(TRIOL^NO_2_^)_2_]^−3^_,_ respectively.

Having experimentally determined the p*K*_a_ and *E*_1/2_ for **V**_**6**_**O**_**11**_**(OH)**_**2**_^**–2**^, the BDFE of surface-bound H atoms was calculated to be 65.3 kcal mol^–1^ using the Bordwell equation (eq 1). The BDFE(O–H)_avg_ value obtained describes the average free energy for the transfer of 2e-/2H^+^ from **V**_**6**_**O**_**11**_**(OH)**_**2**_^**–2**^, resulting in the formation of **V**_**6**_**O**_**13**_^**–2**^.

During the collection of the data presented above, an alternative electrochemical method for the determination of BDFE(E–H) of substrates in nonaqueous systems was published by Mayer and co-workers (note that this methodology was adapted from a technique originally presented by Roberts and Bullock^28^).^29^ By measuring the open circuit potential (OCP) of any chemically reversible redox process at equilibrium over a range of concentrations, one can extrapolate the thermodynamics of E–H bond formation for the overall PCET reaction. The OCP can then be directly translated to BDFE(E–H) according to eq 2:
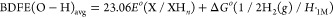
2where *E*^*o*^(X/XH) is equal to the open circuit potential for a 1:1 ratio of reduced and oxidized cluster and Δ*G*^*o*^ is a constant related to the free energy required to homolytically cleave H_2_ in a given solvent.

**Figure 3:**
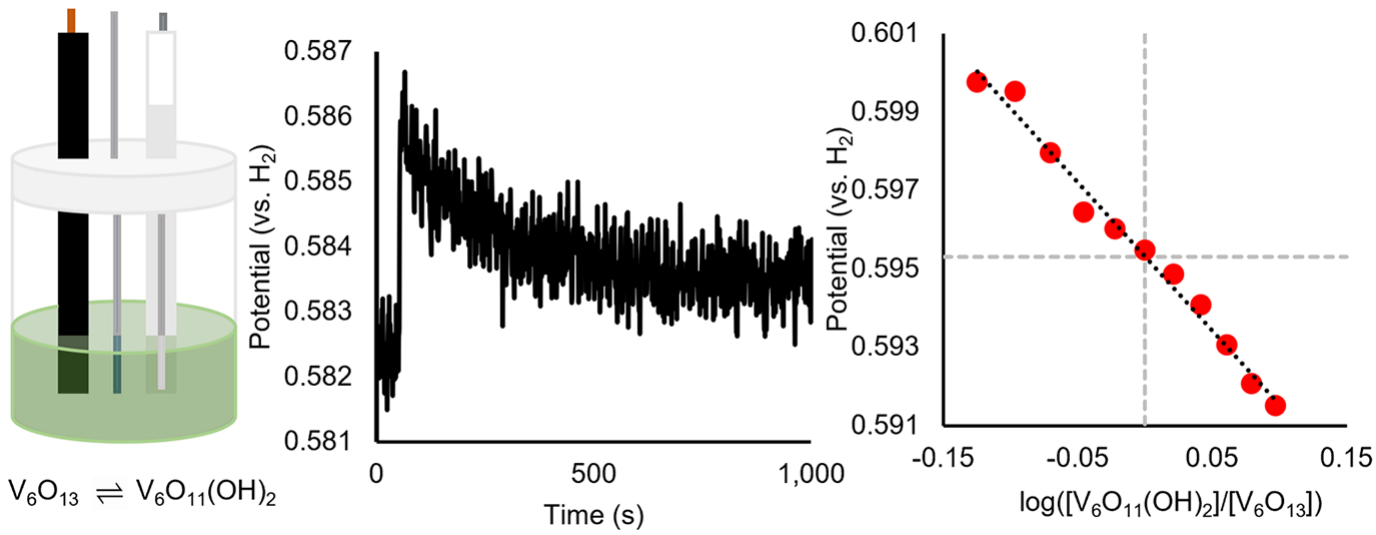
Determination of BDFE(O–H)_avg_ of **V**_**6**_**O**_**11**_**(OH)**_**2**_^**–2**^ using OCP measurements of mixtures of **V**_**6**_**O**_**11**_**(OH)**_**2**_^**–2**^ and **V**_**6**_**O**_**13**_^**–2**^, in 50 mM DMA/DMAH^+^ buffer and 100 mM [^*n*^Bu_4_N][PF_6_] supporting electrolyte in acetonitrile: general experimental setup (left), raw OCP data (middle), and plot of OCP vs log([**V**_**6**_**O**_**11**_**(OH)**_**2**_^**–2**^]/[**V**_**6**_**O**_**13**_^**–2**^]) (right). Portions of this figure are reproduced with permission from ref (1). Copyright 2023 American Chemical Society.

The OCP analytical method was used by our research team to confirm the BDFE(O–H)_avg_ of **V**_**6**_**O**_**11**_**(OH)**_**2**_^**–2**^ (Figure 3). For these measurements, an electrochemical cell was prepared containing *N*,*N*-dimethylaniline/*N*,*N*-dimethylanilinium tetrafluoroborate (DMA/DMAH^+^) buffer and supporting electrolyte in acetonitrile. The DMA/DMAH^+^ buffer was selected based on its ability to facilitate the desired 2e^–^/2H^+^ transfer to the cluster surface; the p*K*_a_ value of DMAH^+^ (11.5) corresponds to the region of potential-p*K*_a_ diagram hypothesized to result in 2e^–^/2H^+^ transfer.

A plot of the measured OCPs against the log of the ratio of concentration of **V**_**6**_**O**_**11**_**(OH)**_**2**_^**–2**^ and its oxidized counterpart **V**_**6**_**O**_**13**_^**–2**^ yields the expected Nernstian dependence for a 2e^–^/2H^+^ transfer (−0.037 ± .0033 V dec^–1^). When referenced against the H^+^/H_2_ couple, the OCP for a 1:1 ratio of reduced and oxidized cluster is 0.595 V (*y*-intercept in Figure 3). The BDFE(O–H)_avg_ value for **V**_**6**_**O**_**11**_**(OH)**_**2**_^**–2**^ was calculated to be 65.7 kcal mol^–1^, according to eq 3:
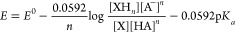
3where *E*^*o*^(X/XH) is the *y*-intercept of our plot and Δ*G*^*o*^ is 52 kcal mol^–1^ in acetonitrile.

The final approach employed for determining the BDFE(O–H)_avg_ value for **V**_**6**_**O**_**11**_**(OH)**_**2**_^**–2**^ involves chemical benchmarking with substrates of known BDFE(E–H) values. This strategy is widely used, as it invokes the equilibrium state between substrate and sample as a direct measure of bond strength.^9,30^ The extent of reactivity between the reduced cluster, **V**_**6**_**O**_**11**_**(OH)**_**2**_^**–2**^, and (2,2,6,6,-tetramethylpiperidin-1-yl)oxyl (TEMPO) was measured via electronic absorbance spectroscopy. The reduced POV-alkoxide possesses a distinct intervalence charge transfer band that is quenched upon oxidation to the isovalent, “V^V^_6_” assembly, allowing for the concentration of the reduced species in solution to be determined. Using the BDFE(O–H) measured for TEMPOH in acetonitrile (BDFE(O–H) = 66 kcal mol^–1^), a BDFE(O–H)_avg_ of 66.1 kcal mol^–1^ was calculated for **V**_**6**_**O**_**11**_**(OH)**_**2**_^**–2**^ via eq 4:
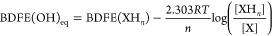
4where BDFE(O–H)_eq_ is the BDFE of the cluster at equilibrium and BDFE(XH_*n*_) is the reported BDFE(O–H) for TEMPOH, *R* is the gas constant, *T* is temperature, *n* is the number of H atoms transferred to 1 equiv of TEMPO (*n* = 1) and [XH_*n*_] and [X] are the measured concentrations of reduced and oxidized the substrate in solution.

**Figure 4:**
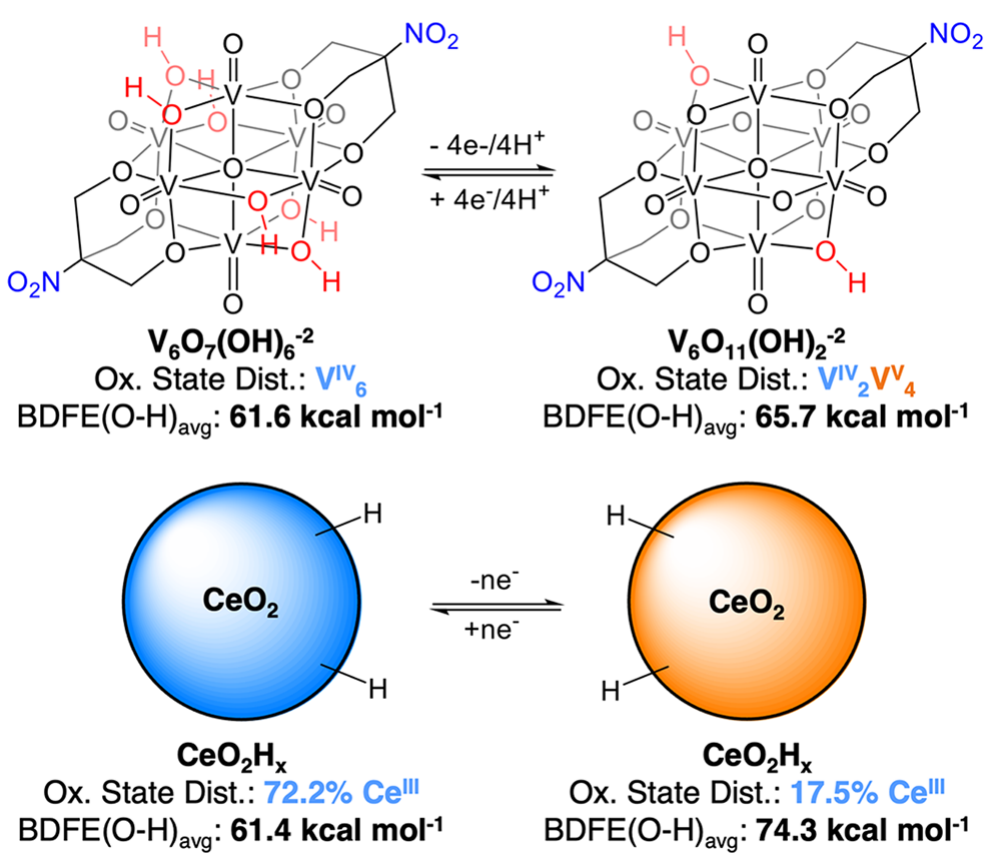
Dependence of BDFE(O–H)_avg_ on the degree of reduction of POV-alkoxide clusters (top) and oleate-capped ceria nanocrystals,^9^ represented by the number of reduced metal centers comprising the material.

The results summarized above confirm analogous BDFE(O–H)_avg_ values for **V**_**6**_**O**_**11**_**(OH)**_**2**_^**–2**^ through a variety of experimental techniques. While any one of these methods are reliable for the determination of thermochemical descriptors of PCET at POM surfaces, our group has found favor with the OCP analysis method. We have since applied OCP analysis to evaluate BDFE(O–H)_avg_ of the most reduced variant of the series summarized in Scheme 2, **V**_**6**_**O**_**7**_**(OH)**_**6**_^**–2**^ (Figure 4). The OCP was measured for a series of solutions containing various ratios of **V**_**6**_**O**_**7**_**(OH)**_**6**_^**–2**^ and its oxidized counterpart, **V**_**6**_**O**_**9**_**(OH)**_**4**_^**–2**^, along with supporting electrolyte and excess 1,1,3,3-tetramethylguanidine/1,1,3,3-tetramethylguanidinium tetrafluoroborate (p*K*_a_(TMG/TMGH^+^) = 23.35 in acetonitrile). It is important to note the use of a different buffer in these OCP analyses; a weaker acid was selected due to the increased basicity of the reduced POV-alkoxide surface.^1^ Results revealed a 2e^–^/2H^+^ transfer from **V**_**6**_**O**_**7**_**(OH)**_**6**_^**–2**^, with a BDFE(O–H)_avg_ of 61.6 kcal mol^–1^.

Comparison of the BDFE(O–H)_avg_ values measured for **V**_**6**_**O**_**11**_**(OH)**_**2**_^**–2**^ and **V**_**6**_**O**_**7**_**(OH)**_**6**_^**–2**^ reveal that as electrons are added to the assembly, more reactive H atom equivalents are stored at the surface. This observation suggests that oxidation state distribution of vanadium ions contained within the Lindqvist ion is a key factor influencing O–H bond strength. A similar oxidation state dependence has been observed in metal oxide nanomaterials, where the BDFE (O–H) of surface hydroxides are demonstrated to be dependent upon the extent of reduction of the nanoparticles.^9^ In this report, a 13 kcal mol^–1^ change in BDFE(O–H)_avg_ for surface-bound H atoms on ceria was accomplished by tuning the ratio of Ce^III^/Ce^IV^ in the material from 17.5 to 72.2%. This is a substantially greater change than the 0.6 kcal mol^–1^ predicted by the Nernst equation. Similarly, the 4 kcal mol^–1^ change in BDFE(O–H)_avg_ observed between **V**_**6**_**O**_**11**_**(OH)**_**2**_^**–2**^ and **V**_**6**_**O**_**7**_**(OH)**_**6**_^**–2**^ is 5 times greater than predicted. This is reasonable considering the Robin and Day Class II delocalized electronic structure of the mixed-valent Lindqvist complexes along the 6e^–^/6H^+^ reduction scheme, meaning electronic delocalization in the core is thermally limited. Partial charge localization in both systems results in deviations to M–O bond geometries (e.g., bond lengths and angles), resulting in nonidealized, chemically distinct surface sites which break the predicted Nernstian dependence of BDFE on electronic occupancy.^9^

The BDFE(O–H)_avg_ values of **V**_**6**_**O**_**11**_**(OH)**_**2**_^**–2**^ and **V**_**6**_**O**_**7**_**(OH)**_**6**_^**–2**^ are low in comparison to reported values of O–H bond strengths in transition-metal oxide clusters. Tilley and co-workers investigated the thermochemistry of H atom uptake at the surface of a high-valent cobalt oxide cubane.^31^ The BDFE(O–H) value of the 1e^–^/1H^+^ reduced assembly was determined through theoretical analysis to be 74 kcal mol^–1^; the authors confirmed this value by probing the reactivity of the assembly toward C–H bond activation. Doerrer and co-workers reported similar reactivity in a divanadium oxide complex, [(CH_3_)_4_][V_2_(O)_2_(μ-O)_2_(pin^F^)]; while explicit determination of the BDFE(O–H)_avg_ value for the 2e^–^/2H^+^ reduced species was not determined, the reactivity described in the paper suggests formation of strong O–H bonds at these bridging oxide sites (>70 kcal mol^–1^). The divanadium substituted polyoxotungsate cluster discussed previously has a significantly higher BDFE(O–H) (75.4 kcal mol^–1^). The lability of the H atom equivalents stored at the surface of **V**_**6**_**O**_**11**_**(OH)**_**2**_^**–2**^ and **V**_**6**_**O**_**7**_**(OH)**_**6**_^**–2**^ renders these clusters suitable to serve as H atom transfer reagents in small molecule hydrogenation reactions. While beyond the scope of this Account, we direct interested readers to work describing mechanistic analysis of the reduction O_2_ by **V**_**6**_**O**_**7**_**(OH)**_**6**_^**–2**^.^1^

### PCET at Terminal Oxides in Polyoxovanadates

**Figure 5:**
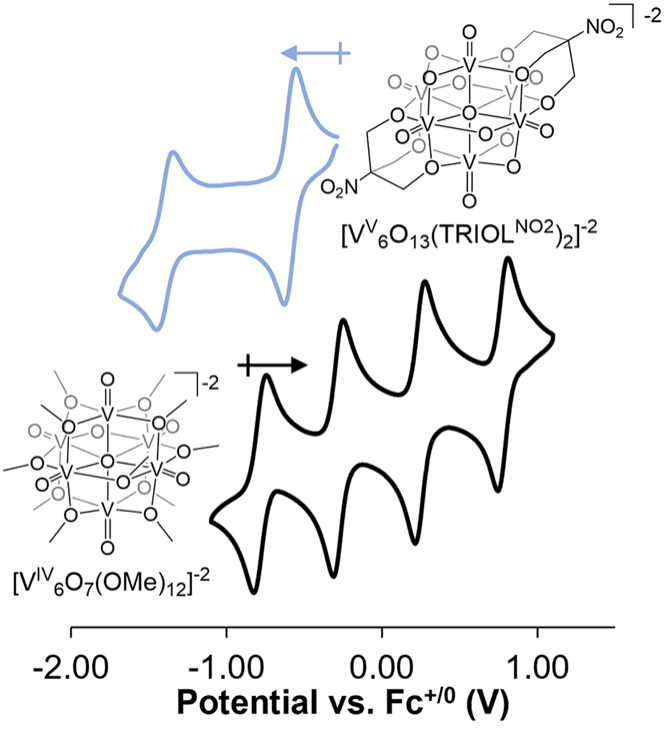
Comparison of the CVs of [V_6_O_13_(TRIOL^NO_2_^)_2_]^−2^ and [V_6_O_7_(OMe)_12_] in MeCN with 100 mM [^*n*^Bu_4_N][PF_6_] as the supporting electrolyte.

While bridging oxide ligands have been shown to be integral to facilitating net H atom transfer reactions at the surface of POMs,^1,2,18^ there is a lack of research probing similar phenomena at terminal oxides.^3,4,22,23^ Accordingly, our research team has studied PCET in a series of reduced, Lindqvist-type POV-alkoxide clusters, [V_6_O_7_(OR)_12_]*^n^* (R = Me, Et); saturation of bridging positions stabilizes a range of charge states as evident in CV (*n* = −2, −1, 0, +1; Figure 5).^32^ Complete functionalization of the POV surface additionally serves to block the nucleophilic bridging sites of the cluster, directing PCET to surface V=O moieties.

#### Proton Uptake in POV-Alkoxide Clusters

**Figure 6:**
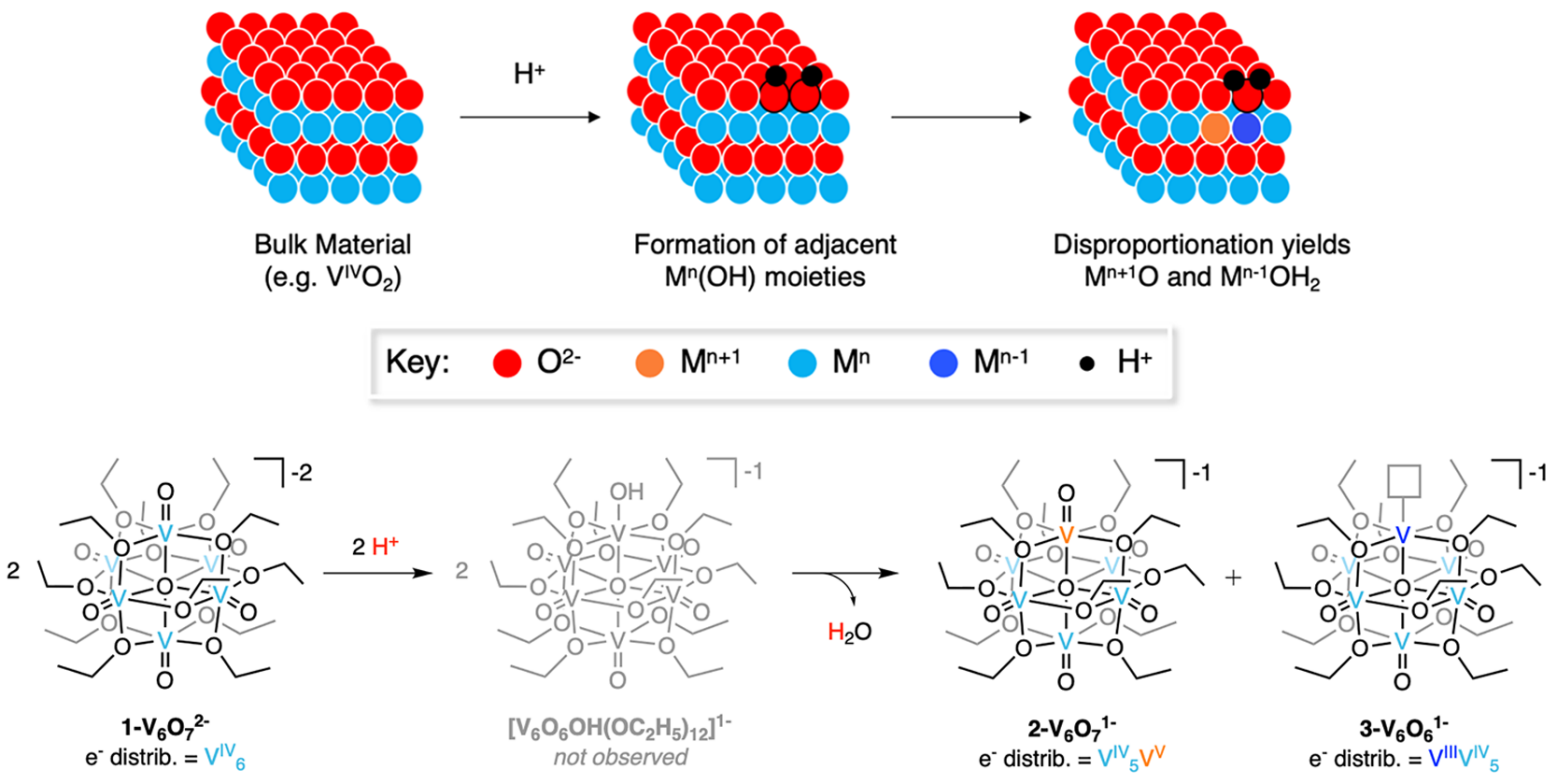
Schematic representation of proton-induced activation of a metal oxide surface (top), and the comparable reactivity observed at V^IV^=O sites in POV-ethoxide clusters (bottom). Figure and caption are reproduced with permission from ref (22). Copyright 2020 American Chemical Society.

Toward understanding PCET at organosaturated POV-alkoxide clusters, initial work probed the reactivity of the fully reduced cluster with protons.^3,22^ Addition of 1 equiv of triethylammonium tetrafluoroborate to [V_6_O_7_(OEt)_12_]^−2^ results in a 1:1 mixture of products, namely [V_6_O_7_(OEt)_12_]^−1^ and [V_6_O_6_(MeCN)(OEt)_12_]^−1^. The ability to access an O atom-deficient POV-alkoxide (i.e., [V_6_O_6_(MeCN)(OEt)_12_]^−1^) through the addition of acid is broadly reminiscent of the surface acid/base chemistry of bulk metal oxides (Figure 6).^33^ Protonation of a terminal M^*n*^=O moiety produces an unstable, M*^n^*–OH which may disproportionate to form M^*n*+1^=O and M^*n*–1^–OH_2_ sites at the surface. We propose a similar mechanism is operative in the case of POV-alkoxide clusters; protonation of [V_6_O_7_(OEt)_12_]^−2^ results in the transient formation of [V_6_O_6_(OH)(OEt)_12_]^−1^, which subsequently disproportionates to generate the 1:1 mixture of [V_6_O_7_(OEt)_12_]^−1^ and [V_6_O_6_(OH_2_)(OEt)_12_]^−1^. The aquo-ligated reduced assembly loses OH_2_ in favor of solvent (MeCN) coordination. Analogous reactivity is observed in the case of the methoxide-bridged species, [V_6_O_7_(OMe)_12_]^−2^, which has been used in subsequent reporting on proton and H atom uptake mechanisms.

**Figure 7:**
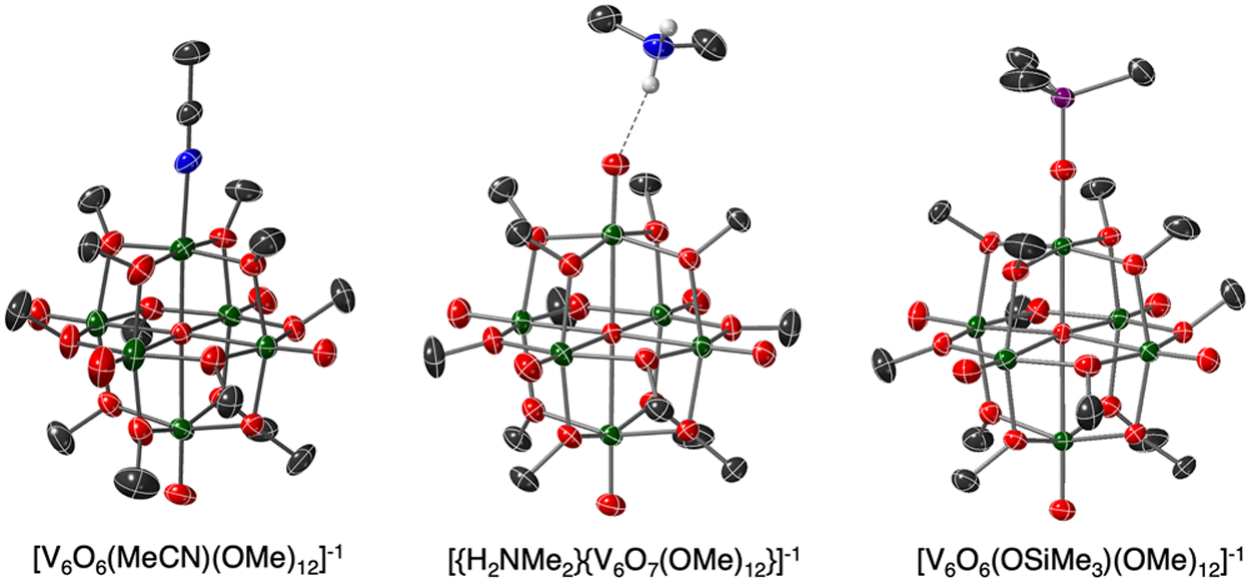
Molecular structures of [^*n*^Bu_4_N][V_6_O_6_(MeCN)(OMe)_12_] (left), [H_2_NMe_2_][^*n*^Bu_4_N][V_6_O_7_(OMe)_12_] (middle) and [^*n*^Bu_4_N][V_6_O_6_(OSiMe_3_)(OMe)_12_] (right) shown with 30% probability ellipsoids. Counterions, solvent molecules, and selected H atoms removed for clarity. Key: dark green ellipsoids, V; red ellipsoids, O; dark gray ellipsoids, C; blue ellipsoids, N; purple ellipsoids, Si; white spheres, H. Figure was reproduced with permissions from refs (2, 3, and 16). Copyright 2021 and 2022 American Chemical Society and 2021 Royal Society of Chemistry.

**Scheme 3:**
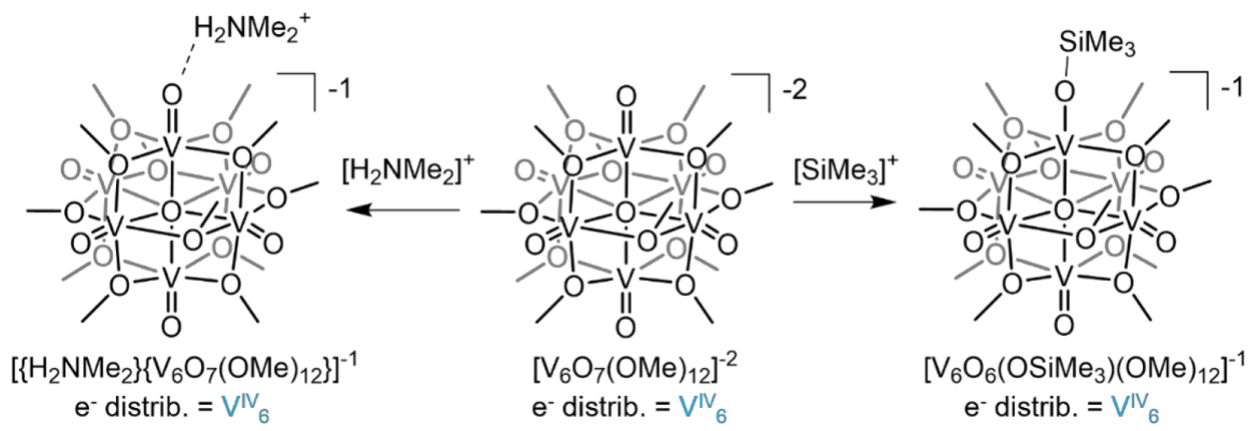
Mechanistic Investigations Supporting Terminal Hydroxide Formation En Route to Proton-Induced O Atom Defect Formation at V^IV^=O Moieties

We note that the proposed hydroxide intermediate, [V_6_O_6_(OH)(OR)_12_]^−1^, has not been directly observed despite exhaustive attempts to characterize this species. However, evidence in support of the mechanism in Figure 6 has been surmised through a series of experiments (Scheme 3). During investigation of the reactivity of [V_6_O_7_(OMe)_12_]^−2^ with dimethylammonium chloride, analysis of crystals grown serendipitously from the reaction mixture revealed formation of an ion pair with hydrogen bonding between a proton of the cation and a terminal oxo (Figure 7).^3^ Additional evidence was found in studies involving the use of silylium ions as a bulky proton surrogate; exposure of trimethylsilyltrifluoromethylsulfonate to [V_6_O_7_(OMe)_12_]^−2^ at low temperature results in quantitative conversion to the siloxide species, [V_6_O_6_(OSiMe_3_)(OMe)_12_]^−1^ (Figure 7).^24^

Subsequently our team interrogated the dependence of cluster oxidation state on the basicity of the vanadium oxide assembly.^3^ Toward this goal, we probed the reactivity of [V_6_O_7_(OMe)_12_]^−2^ (V^IV^_6_), [V_6_O_7_(OMe)_12_]^−1^ (V^IV^_5_V^V^) and [V_6_O_7_(OMe)_12_]^0^ (V^IV^_4_V^V^_2_) with a series of organic acids with p*K*_a_ values ranging from 1.28 to 22.6 in MeCN. In all charge states, protonation of [V_6_O_7_(OMe)_12_]^*n*^ produces the corresponding oxidized [V_6_O_7_(OMe)_12_]^*n*+1^ and oxygen-deficient POV-alkoxides, [V_6_O_6_(MeCN)(OMe)_12_]^*n*+1^. Monitoring the concentration of products formed allows us to approximate the p*K*_a_ across the redoxomer series. These results indicate that oxidation of the cluster decreases the basicity of the surface by approximately 7 p*K*_a_ units per electron. These results reveal how core electron density supersedes overall charge in influencing proton uptake.

#### Hydrogen Atom Uptake in POV-Alkoxide Clusters

**Scheme 4:**
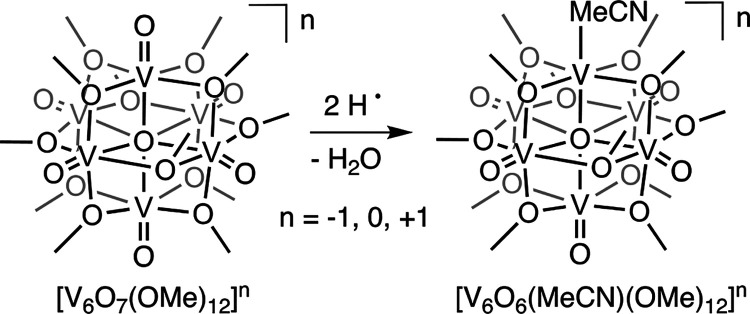
O Atom Defect Formation via H Atom Transfer to Terminal V^V^=O Sites on POV-Alkoxides Scheme reproduced with permission from ref (4). Copyright 2022 American Chemical Society.

The observed dependence of surface basicity of POV-alkoxide clusters on cluster oxidation state inspired studies into the uptake of H atom equivalents at terminal vanadyl sites. As such, we investigated the reactivity of [V_6_O_7_(OMe)_12_]^−1^ with 5,10-dihydrophenazine (H_2_Phen, BDFE(N–H)_avg_ = 58.7 kcal mol^–1^^8^).^36^ Upon addition of 1 equiv of this substrate to [V_6_O_7_(OMe)_12_]^−1^ in MeCN, quantitative formation of the O atom-deficient POV-alkoxide cluster [V_6_O_6_(MeCN)(OMe)_12_]^−1^ was observed alongside phenazine and water (Scheme 4).

The transfer of hydrogen atoms in a chemical reaction is dependent on the relative strengths of E–H bonds involved, rendering quantification of E–H bond strengths important for the prediction of reactivity. In the prior section, we established a series of experimental approaches to determine BDFE(O–H) values of surface-bound H atoms. Unfortunately, due to the irreversible reactivity of the [V_6_O_7_(OCH_3_)_12_]^*n*^ family of clusters with acid, BDFE(O–H) values cannot be measured electrochemically. As such, we relied on chemical benchmarking experiments with H atom donors with known BDFE(E–H) values to map the reactivity of the [V_6_O_7_(OCH_3_)_12_]^−1^. The selected substrates included hydrazobenzene (Hydz; BDFE(N–H)_avg_ = 60.9 kcal mol^–1^), 2,6-ditertbutyl-1,4-hydroquinone (^*t*^Bu_2_HQ, BDFE(O–H)_avg_ = 62.8 kcal mol^–1^), 2,6-dimethyl-1,4-hydroquinone (Me_2_HQ; BDFE(O–H)_avg_ = 64.6 kcal mol^–1^), and 1,4-hydroquinone (HQ, BDFE(O–H) = 67.3 kcal mol^–1^).^8^ No reaction was observed between [V_6_O_7_(OCH_3_)_12_]^−1^ and the hydroquinone substrates, indicating their O–H bonds are too strong for appreciable H atom transfer to take place. However, partial conversion to the O atom-deficient POV-alkoxide was observed upon exposure of [V_6_O_7_(OMe)_12_]^−1^ to Hydz, suggesting the bond strength of [V_6_O_6_(OH)(OMe)_12_]^−1^ is between 60 and 62 kcal mol^–1^.

Our attention then turned to kinetic analysis to glean mechanistic details about H atom transfer to the surface of the POV-alkoxide cluster. Pseudo-first-order reactions between [V_6_O_7_(OMe)_12_]^−1^ and H_2_Phen revealed a bimolecular rate-limiting step between cluster and reductant. Comparing the reaction rate observed with D_2_Phen reveals a kinetic isotope effect (KIE) of 2.1, suggesting proton movement is involved in the rate-determining step of the reaction. Evidence invoking a CPET mechanism for H atom uptake at [V_6_O_7_(OMe)_12_]^−1^ was obtained by measuring the temperature dependence on the rate of defect formation at the surface of the assembly. Activation parameters (e.g., Δ*H*^⧧^, Δ*S*^⧧^, and Δ*G*^⧧^) were obtained via Eyring analysis; a large negative value of Δ*S*^⧧^ (−40.9 ± 3 cal mol^–1^ K^–1^) is observed, consistent with the rate-limiting step of the reaction proceeding through a highly ordered transition state. We hypothesize that this involves a hydrogen bond between the H atom donor (H_2_Phen) and acceptor ([V_6_O_7_(OMe)_12_]^−1^). Similar values of Δ*S*^⧧^ have been reported for hydrogen-bonded donor–acceptor pairs generated during the rate-determining step of CPET reactions.^1,2,34^

**Scheme 5:**
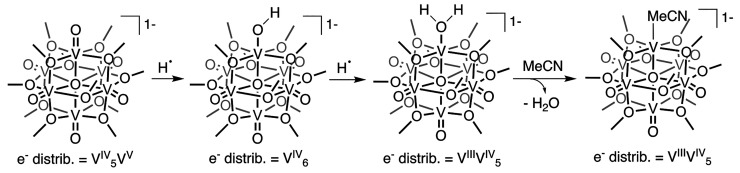
Proposed Mechanism for the Formation of [V_6_O_6_(MeCN)(OMe)_12_]^−1^ via PCET

With these results in hand, a mechanism of defect formation via H atom uptake is proposed (Scheme 5).^36^ A rate-determining CPET step from H_2_Phen to [V_6_O_7_(OMe)_12_]^−1^ results in formation of [V_6_O_6_(OH)(OMe)_12_]^−1^, followed by the rapid transfer of the second H atom equivalent from the organic reagent to generate [V_6_O_6_(OH_2_)(OMe)_12_]^−1^. When this reaction is conducted in MeCN, the aquo ligand is displaced, giving rise to the final product, [V_6_O_6_(MeCN)(OMe)_12_]^−1^. We find that this reactivity curtails the disproportionation step that follows protonation of the terminal oxo, as the second N–H bond is expected to be significantly weaker,^8^ providing driving force for the second H atom equivalent to be directly installed by the 1e^–^/1H^+^ oxidized hydrophenazyl radical. This distinguishes the reactivity of the organic-saturated POV-alkoxides with H atom versus proton donors, as the ready availability of a second H atom equivalent curtails disproportionation of transiently formed terminal hydroxide-containing species.

Additional support for the proposed mechanism outlined in Scheme 5 is derived from the reactivity of POV-alkoxide clusters with Mashima’s reagent (1,4-bis(trimethylsilyl)-1,4,-diaza-2,5-cyclohexadiene, Pyz(SiMe_3_)_2_).^35^ This substrate has been popularized as a metal-free reductant, efficiently activating metal oxides via silyl radical transfer, and more recently has been invoked as a model for H atom transfer reactions.^35,36^ Addition of 1/2 equiv of Pyz(SiMe_3_)_2_ to [V_6_O_7_(OMe)_12_]^−1^ at low temperature afforded quantitative formation of the siloxide-functionalized POV-alkoxide, [V_6_O_6_(OSiMe_3_)(OMe)_12_]^−1^. The product serves as an excellent model of the 1e^–^/1H^+^ reduced species, whose formation is proposed following the rate-determining step of O atom vacancy formation via H atom uptake (Scheme 5).

**Figure 8:**
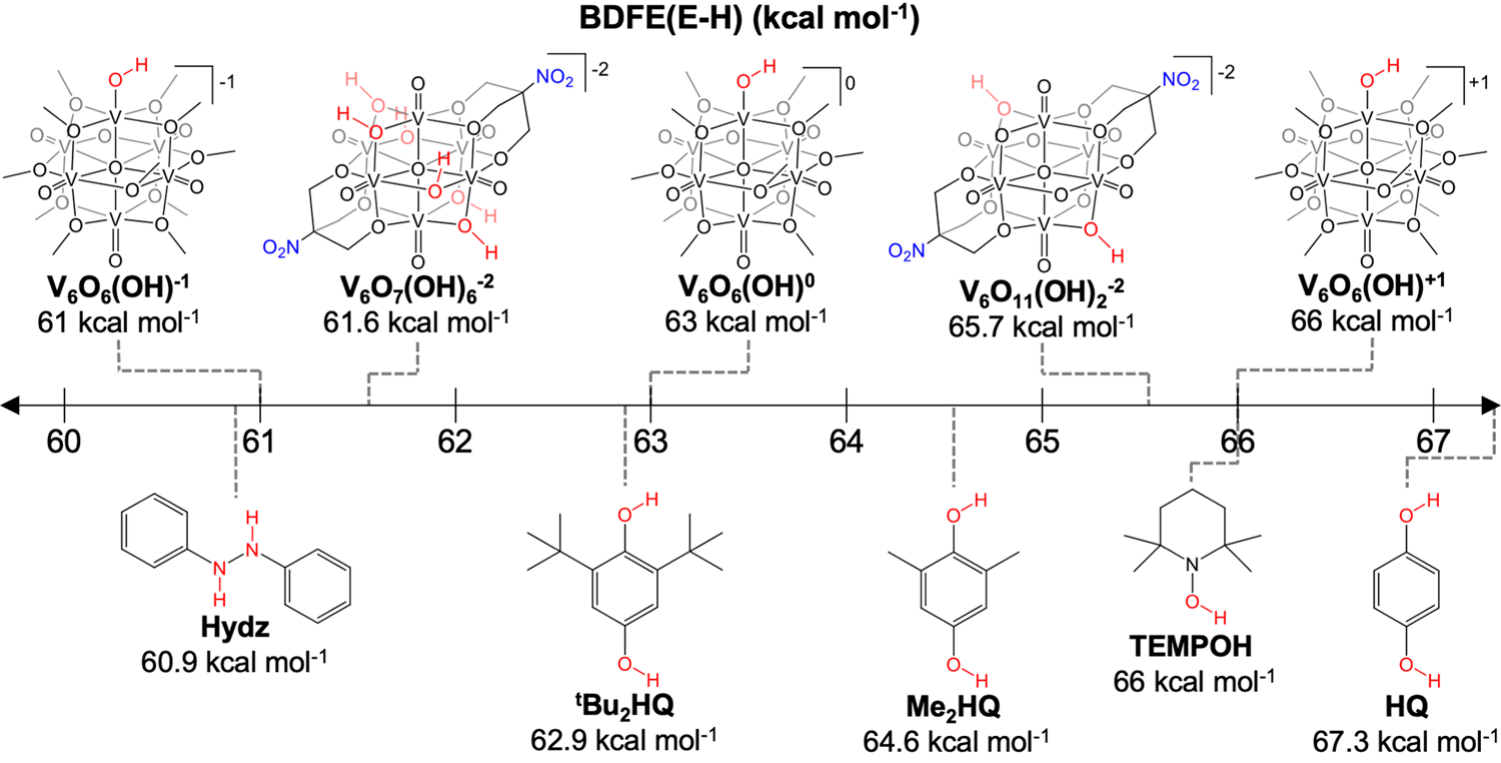
Comparison of the BDFE(O–H)_avg_ determined for the reduced POV-alkoxides described in this Account^1,2,4^ with a variety of organic PCET reagents.^8^

As the oxidation state distribution of distal vanadium ions was observed to have a dramatic impact on the basicity of the cluster surface, we hypothesized that charge state of the assembly would influence the thermodynamics of H atom uptake. Similar to the experiments described above targeting elucidation of the BDFE(O–H) for [V_6_O_6_(OH)(OMe)_12_]^−1^, the reactivity of [V_6_O_7_(OMe)_12_]^0^ and [V_6_O_7_(OMe)_12_]^+1^ with H atom transfer reagents was explored.^36^ The more-oxidized vanadium oxide assemblies are able to abstract H atoms from substrates with higher BDFE(E–H) values. Based on the observed reactivity, we approximated the BDFE(O–H) of [V_6_O_6_(OH)(OMe)_12_]^0^ to be ∼63 kcal mol^–1^, and the BDFE(O–H) of [V_6_O_6_(OH)(OMe)_12_]^+1^ to be ∼66 kcal mol^–1^ (Figure 8). This series of results indicates that the oxidation state distribution of distal vanadium centers influences the affinity of a single vanadium oxide site for H atoms, similar to that observed at bridging oxide sites (*vide supra*).^4^

## Conclusions and Outlook

In this Account, we have summarized our efforts to provide atomic insight into PCET reactivity at metal oxide surfaces using POV-alkoxide clusters. The work described predominantly focuses on the quantification of the free energy of O–H bonds formed at the surface of reduced vanadium oxide assemblies. Structural modifications to the POV-alkoxide precursor allow our research team to interrogate differences in thermodynamics of O–H bonds generated at terminal- and bridging-oxide sites, providing insight into the regioselectivity of net H atom uptake at transition-metal oxide surfaces.

The bridging oxides of high-valent, Lindqvist-type POV-alkoxide clusters are able to store up to 6 proton/electron pairs at the surface of the assembly. Net H atom uptake and transfer occurs pairwise in these systems, with isolable forms of the reduced assembly differing by 2 H atom equivalents (e.g., [V_6_O_13_(TRIOL^NO_2_^)_2_]^−2^, [V_6_O_11_(OH)_2_(TRIOL^NO_2_^)_2_]^−2^, [V_6_O_9_(OH)_4_(TRIOL^NO_2_^)_2_]^−2^, [V_6_O_7_(OH)_6_(TRIOL^NO_2_^)_2_]^−2^). Thermochemical descriptors (i.e., BDFE(O–H)_avg_ values) for each of the reduced clusters were determined using several methods presented in literature. Our studies reveal weak H atom interactions at the surface of the POV-alkoxide cluster, with BDFE(O–H)_avg_ values ranging from 61.6 to 65.7 kcal mol^–1^. Additionally, the affinity of proton/electron pairs for the surface of the cluster is correlated to the oxidation state distribution of vanadium ions; BDFE(O–H)_avg_ values describing the transfer of H atoms from POV-alkoxides are lower as the cluster core becomes further reduced.

Complementary investigations have probed net H atom uptake and transfer at terminal oxide sites in [V_6_O_7_(OR)_12_]^*n*^ (R = Me, Et; *n* = −2, −1, 0, +1). Saturation of bridging positions with alkoxide moieties inhibits the interaction of proton and H atom equivalents with bridging oxygen atoms. Addition of H atom equivalents to the surface of the assembly results in reduction of V^V^=O moieties to V^III^–OH_2_. Mechanistic investigations reveal that net H atom uptake occurs via a series of CPET-type reactions. Our experiments reveal BDFE(O–H) values for purported hydroxide-substituted intermediates, [V_6_O_6_(OH)(OMe)_12_]^*n*^, range between 60 and 66 kcal mol^–1^; with the lability of surface H atoms similarly correlated to the oxidation state distribution (i.e., core electron density) of vanadium ions.

Our investigations quantifying thermodynamic and kinetic properties of PCET in POV-alkoxide clusters at disparate oxido ligands provide a launch point for future mechanistic work. Our findings indicate comparable thermodynamic driving forces for H atom installation at these disparate sites, suggesting selectivity for a particular type of oxide moiety may be governed by kinetics of the relevant PCET reactions. Recent work has demonstrated the ability to modify PCET pathways for a given substrate through changes in molecular structure and reaction environment.^37^ Such mechanistic changes can result in increased selectivity for small molecule activation. Ongoing investigations by our research team aim to develop an understanding of how modifications to the structure of the POV-alkoxide influence the mechanism of PCET at the surface of the cluster. Promising preliminary results show striking variations in kinetic isotope effects and activation parameters, indicating that fundamental concepts understood in discrete monometallic catalysts for net H atom transfer can also be leveraged to control the reactivity in multimetallic metal oxide assemblies.

## References

[ref1] Fertig A. A.; Matson E. M. Connecting Thermodynamics and Kinetics of Proton Coupled Electron Transfer at Polyoxovanadate Surfaces Using the Marcus Cross Relation. Inorg. Chem. 2023, 62, 1958–1967. 10.1021/acs.inorgchem.2c02541. 36049052 PMC9906739

[ref2] Fertig A. A.; Brennessel W. W.; McKone J. R.; Matson E. M. Concerted Multiproton-Multielectron Transfer for the Reduction of O_2_ to H_2_O with a Polyoxovanadate Cluster. J. Am. Chem. Soc. 2021, 143 (38), 15756–15768. 10.1021/jacs.1c07076. 34528799

[ref3] Schreiber E.; Brennessel W. W.; Matson E. M. Charge-State Dependence of Proton Uptake in Polyoxovanadate-alkoxide Clusters. Inorg. Chem. 2022, 61, 4789–4800. 10.1021/acs.inorgchem.1c02937. 35293218 PMC8965876

[ref4] Schreiber E.; Fertig A. A.; Brennessel W. W.; Matson E. M. Oxygen-Atom Defect Formation in Polyoxovanadate Clusters via Proton-Coupled Electron Transfer. J. Am. Chem. Soc. 2022, 144, 5029–5041. 10.1021/jacs.1c13432. 35275632 PMC8949770

[ref5] aShaheen K.; Shah Z.; Gulab H.; Hanif M. B.; Faisal S.; Suo H. Metal oxide nanocomposites as anode and cathode for low temperature solid oxide fuel cell. Solid State Sci. 2020, 102, 106162 10.1016/j.solidstatesciences.2020.106162.

[ref6] Baucke F. G. K. Electrochromic mirrors with variable reflectance. Solar Energy Materials 1987, 16, 67–77. 10.1016/0165-1633(87)90009-8.

[ref7] Khan M. M.; Adil S. F.; Al-Mayouf A. Metal oxides as photocatalysts. Journal of Saudi Chemical Society 2015, 19, 462–464. 10.1016/j.jscs.2015.04.003.

[ref8] Agarwal R. G.; Coste S. C.; Groff B. D.; Heuer A. M.; Noh H.; Parada G. A.; Wise C. F.; Nichols E. M.; Warren J. J.; Mayer J. M. Free Energies of Proton-Coupled Electron Transfer Reagents and Their Applications. Chem. Rev. 2022, 122, 1–49. 10.1021/acs.chemrev.1c00521. 34928136 PMC9175307

[ref9] Agarwal R. G.; Kim H.-J.; Mayer J. M. Nanoparticle O–H Bond Dissociation Free Energies from Equilibrium Measurements of Cerium Oxide Colloids. J. Am. Chem. Soc. 2021, 143, 2896–2907. 10.1021/jacs.0c12799. 33565871

[ref10] aWise C. F.; Mayer J. M. Electrochemically Determined O–H Bond Dissociation Free Energies of NiO Electrodes Predict Proton-Coupled Electron Transfer Reactivity. J. Am. Chem. Soc. 2019, 141, 14971–14975. 10.1021/jacs.9b07923. 31513390

[ref11] aFleischmann S.; Mitchell J. B.; Wang R.; Zhan C.; Jiang D.-e.; Presser V.; Augustyn V. Pseudocapacitance: From Fundamental Understanding to High Power Energy Storage Materials. Chem. Rev. 2020, 120, 6738–6782. 10.1021/acs.chemrev.0c00170. 32597172

[ref12] aHermann K.; Witko M.; Druzinic R. Electronic properties, structure and adsorption at vanadium oxide: density functional theory studies. Faraday Discuss. 1999, 114, 53–66. 10.1039/a903109j.

[ref13] aMiu E. V.; McKone J. R.; Mpourmpakis G. The Sensitivity of Metal Oxide Electrocatalysis to Bulk Hydrogen Intercalation: Hydrogen Evolution on Tungsten Oxide. J. Am. Chem. Soc. 2022, 144, 6420–6433. 10.1021/jacs.2c00825. 35289172

[ref14] aWarburton R. E.; Mayer J. M.; Hammes-Schiffer S. Proton-Coupled Defects Impact O–H Bond Dissociation Free Energies on Metal Oxide Surfaces. J. Phys. Chem. Lett. 2021, 12, 9761–9767. 10.1021/acs.jpclett.1c02837. 34595925

[ref15] Fujibayashi M.; Watari Y.; Tsunashima R.; Nishihara S.; Noro S. I.; Lin C. G.; Song Y. F.; Takahashi K.; Nakamura T.; Akutagawa T. Structural Phase Transitions of a Molecular Metal Oxide. Angew. Chem., Int. Ed. 2020, 59, 22446–22450. 10.1002/anie.202010748. 32856378

[ref16] Chakraborty S.; Schreiber E.; Sanchez-Lievanos K. R.; Tariq M.; Brennessel W. W.; Knowles K. E.; Matson E. M. Modelling local structural and electronic consequences of proton and hydrogen-atom uptake in VO(2) with polyoxovanadate clusters. Chem. Sci. 2021, 12, 12744–12753. 10.1039/D1SC02809J. 34703561 PMC8494032

[ref17] aPope M. T.; Müller A. Polyoxometalate Chemistry: An Old Field with New Dimensions in Several Disciplines. Angew. Chem., Int. Ed. 1991, 30, 34–48. 10.1002/anie.199100341.

[ref18] Ueda T. Electrochemistry of Polyoxometalates: From Fundamental Aspects to Applications. Chemelectrochem 2018, 5, 823–838. 10.1002/celc.201701170.

[ref19] aWay D. M.; Cooper J. B.; Sadek M.; Vu T.; Mahon P. J.; Bond A. M.; Brownlee R. T. C.; Wedd A. G. Systematic Electrochemical Synthesis of Reduced Forms of the α-[S2Mo18O62]4-Anion1. Inorg. Chem. 1997, 36, 4227–4233. 10.1021/ic970592v. 11670315

[ref20] Pope M. T. Heteropoly and Isopoly Oxometalates; Springer-Verlag, 1983.

[ref21] Chakraborty S.; Petel B. E.; Schreiber E.; Matson E. M. Atomically precise vanadium-oxide clusters. Nanoscale Advances 2021, 3, 1293–1318. 10.1039/D0NA00877J. 36132875 PMC9419539

[ref22] Schreiber E.; Petel B. E.; Matson E. M. Acid-Induced, Oxygen-Atom Defect Formation in Reduced Polyoxovanadate-Alkoxide Clusters. J. Am. Chem. Soc. 2020, 142, 9915–9919. 10.1021/jacs.0c03864. 32433883

[ref23] Cooney S. E.; Fertig A. A.; Buisch M. R.; Brennessel W. W.; Matson E. M. Coordination-induced bond weakening of water at the surface of an oxygen-deficient polyoxovanadate cluster. Chemical Science 2022, 13, 12726–12737. 10.1039/D2SC04843D. 36519047 PMC9645371

[ref24] aLei J.; Yang J. J.; Liu T.; Yuan R. M.; Deng D. R.; Zheng M. S.; Chen J. J.; Cronin L.; Dong Q. F. Tuning Redox Active Polyoxometalates for Efficient Electron-Coupled Proton-Buffer-Mediated Water Splitting. Chemistry 2019, 25, 11432–11436. 10.1002/chem.201903142. 31309625 PMC6851869

[ref25] Shanmugaprabha T.; Selvakumar K.; Vairalakshmi M.; Rajasekaran K.; Sami P. Proton-coupled electron transfer reactions: kinetic studies on the oxidation of dihydroxybenzenes by heteropoly 10-tungstodivanadophosphate in aqueous acidic medium. Transition Metal Chemistry 2015, 40, 197–205. 10.1007/s11243-014-9906-x.

[ref26] Chen Q.; Goshorn D. P.; Scholes C. P.; Tan X. L.; Zubieta J. Coordination compounds of polyoxovanadates with a hexametalate core. Chemical and structural characterization of [V^V^_6_O_13_[(OCH_2_)_3_CR]_2_]^2-^, [V^V^_6_O_11_(OH)_2_[(OCH_2_)_3_CR]_2_], [V^IV^_4_V^V^_2_O_9_(OH)_4_[(OCH_2_)_3_CR]_2_]^2-^, and [V^IV^_6_O_7_(OH)_6_](OCH_2_)_3_CR]_2_]^2-^. J. Am. Chem. Soc. 1992, 114, 4667–4681. 10.1021/ja00038a033.

[ref27] McCarthy B. D.; Dempsey J. L. Decoding Proton-Coupled Electron Transfer with Potential-pK(a) Diagrams. Inorg. Chem. 2017, 56, 1225–1231. 10.1021/acs.inorgchem.6b02325. 28075117

[ref28] Roberts J. A.; Bullock R. M. Direct determination of equilibrium potentials for hydrogen oxidation/production by open circuit potential measurements in acetonitrile. Inorg. Chem. 2013, 52, 3823–3835. 10.1021/ic302461q. 23488870

[ref29] Wise C. F.; Agarwal R. G.; Mayer J. M. Determining Proton-Coupled Standard Potentials and X-H Bond Dissociation Free Energies in Nonaqueous Solvents Using Open-Circuit Potential Measurements. J. Am. Chem. Soc. 2020, 142, 10681–10691. 10.1021/jacs.0c01032. 32432468

[ref30] aLucarini M.; Pedrielli P.; Pedulli G. F.; Cabiddu S.; Fattuoni C. Bond dissociation energies of O-H bonds in substituted phenols from equilibration studies. J. Org. Chem. 1996, 61, 9259–9263. 10.1021/jo961039i.

[ref31] Amtawong J.; Skjelstad B. B.; Balcells D.; Tilley T. D. Concerted Proton–Electron Transfer Reactivity at a Multimetallic Co4O4 Cubane Cluster. Inorg. Chem. 2020, 59, 15553–15560. 10.1021/acs.inorgchem.0c02625. 32997494

[ref32] Spandl J.; Daniel C.; Brudgam I.; Hartl H. Synthesis and structural characterization of redox-active dodecamethoxoheptaoxohexavanadium clusters. Angew. Chem., Int. Ed. 2003, 42, 1163–1166. 10.1002/anie.200390306. 12640650

[ref33] aManjakkal L.; Szwagierczak D.; Dahiya R. Metal oxides based electrochemical pH sensors: Current progress and future perspectives. Prog. Mater. Sci. 2020, 109, 100635 10.1016/j.pmatsci.2019.100635.

[ref34] aSchreiber E.; Brennessel W. W.; Matson E. M. Regioselectivity of concerted proton–electron transfer at the surface of a polyoxovanadate cluster. Chemical Science 2023, 14, 1386–1396. 10.1039/D2SC05928B. 36794190 PMC9906639

[ref35] Chakraborty S.; Matson E. M. Reductive silylation of polyoxovanadate surfaces using Mashima’s reagent. Inorganic Chemistry Frontiers 2021, 8, 4507–4516. 10.1039/D1QI00920F.

[ref36] Chu J.; Carroll T. G.; Wu G.; Telser J.; Dobrovetsky R.; Ménard G. Probing Hydrogen Atom Transfer at a Phosphorus(V) Oxide Bond Using a “Bulky Hydrogen Atom” Surrogate: Analogies to PCET. J. Am. Chem. Soc. 2018, 140, 15375–15383. 10.1021/jacs.8b09063. 30382703

[ref37] aHuang T.; Rountree E. S.; Traywick A. P.; Bayoumi M.; Dempsey J. L. Switching between Stepwise and Concerted Proton-Coupled Electron Transfer Pathways in Tungsten Hydride Activation. J. Am. Chem. Soc. 2018, 140, 14655–14669. 10.1021/jacs.8b07102. 30362720

